# The multifunctional type VI secretion system of *Vibrio parahaemolyticus*: from environmental adaptation to interbacterial competition

**DOI:** 10.1128/aem.01652-25

**Published:** 2025-11-05

**Authors:** Yang Wang, Tiange Ma, Yingwang Ye

**Affiliations:** 1 College of Biological and Food Engineering, Anhui Polytechnic University 162794, Wuhu, China; 2 School of Pharmacy, Anhui College of Traditional Chinese Medicine 634605, Wuhu, China; 3 School of Food and Biological Engineering, Hefei University of Technology 724680 , Hefei, China; Anses, Maisons-Alfort Laboratory for Food Safety, Maisons-Alfort, France

**Keywords:** *Vibrio parahaemolyticus*, type VI secretion system, effectors, regulatory factors

## Abstract

*Vibrio parahaemolyticus* (*V. parahaemolyticus*) is a widespread marine bacterium that has gained prominence as a major seafood-borne pathogen and a primary cause of human gastroenteritis worldwide. The type VI secretion system (T6SS), a highly specialized molecular apparatus that injects effector proteins into competing cells, plays a pivotal role in mediating the pathogenicity, bacterial competitiveness, and environmental adaptability. This review presents an in-depth overview of the T6SS in *V. parahaemolyticus*, addressing its discovery, molecular architecture, genetic organization, and associated effector proteins. In addition, special attention is given to the functional divergence between T6SS1 and T6SS2, particularly in mediating bacterial antagonism and adaptation to environmental stressors. We further examine the complex regulatory frameworks that control these systems, including environmental signals, surface sensing, quorum sensing, and specific regulators. Understanding these mechanisms advances our knowledge of the survival strategies and pathogenic behaviors of *V. parahaemolyticus*.

## INTRODUCTION

*Vibrio parahaemolyticus* (*V. parahaemolyticus*) is a halophilic, gram-negative bacterium predominantly inhabiting marine, estuarine, and coastal environments. It is a common cause of foodborne illness, typically presenting with gastrointestinal symptoms, such as diarrhea, abdominal pain, nausea, and vomiting. In more severe cases, infection may result in dehydration, septicemia, or even fatality (1, 2). Globally, the prevalence of *V. parahaemolyticus*-related outbreaks has risen, reinforcing its status as a critical public health concern and a leading contributor to bacterial food poisoning. It is particularly prevalent in East Asia—most notably Japan and South Korea—and has emerged as the primary causative agent of seafood-related illnesses in North America and parts of Europe, where incidence rates have been increasing annually (3). The pathogenicity of virulent strains is mediated by an arsenal of virulence determinants, including thermostable direct hemolysin (TDH), TDH-related hemolysin (TRH), polar and lateral flagella, proteolytic enzymes (e.g., protease A and enolase), lipopolysaccharides, outer membrane adhesins (e.g., MAM-7), and various secretion systems, such as T3SS1 (type III secretion system 1), T3SS2, and T6SS1 (type VI secretion system 1). Additionally, toxins like PirAB contribute to its infectious potential (3–9). Among these, current evidence suggests that T6SS1, one of the type VI secretion systems in this species, is predominantly found in strains pathogenic to humans or marine animals (8, 9). The T6SS is a contact-dependent molecular apparatus found in a wide range of gram-negative bacteria, enabling the direct translocation of effector proteins into target cells or the surrounding extracellular environment (10, 11). Comparative genomic analyses have revealed that over 25% of gram-negative bacterial genomes encode T6SS gene clusters, with individual species harboring between one and six distinct clusters (12).

Functionally, the T6SS was first characterized in *Vibrio cholerae* in 2006, when key structural components and hallmark substrates were identified through genetic approaches (13). Since its initial discovery, the T6SS has rapidly emerged as a pivotal bacterial weapon system, with its functional characterization expanding across diverse bacterial genera including *Vibrio*, *Pseudomonas*, *Escherichia*, *Burkholderia*, and *Aeromonas*. One of the most conserved and widely studied functions of the T6SS is its role in mediating interbacterial antagonism. It achieves this by delivering antibacterial effectors—such as peptidoglycan-degrading enzymes and membrane-disrupting toxins—into competing bacterial cells, thereby conferring a competitive advantage to the secreting organism (14, 15). Although early research primarily emphasized the role of T6SS in eukaryotic pathogenesis, including immune evasion and host cell modulation (16, 17), subsequent studies have highlighted its broader physiological functions. Notably, T6SSs in certain bacterial species contribute to environmental adaptation, including metal ion acquisition and detoxification processes (18, 19). Moreover, these systems are involved in biofilm development, stress response mechanisms, and complex interspecies interactions (20, 21).

The first report on the T6SS of *V. parahaemolyticus* appeared in 2008, when Boyd et al. identified a complete T6SS gene cluster in the genome of strain RIMD2210633 using a comparative BLAST-based approach (22). Since then, significant advancements have been made in elucidating the functional diversity, effector proteins, and regulatory dynamics of T6SS in *V. parahaemolyticus*. This review consolidates current findings, offering a detailed examination of the system’s architecture, biological roles, and regulatory mechanisms, which are essential for understanding its contributions to pathogenicity and ecological fitness.

## T6SS COMPONENTS AND STRUCTURE

**Fig 1:**
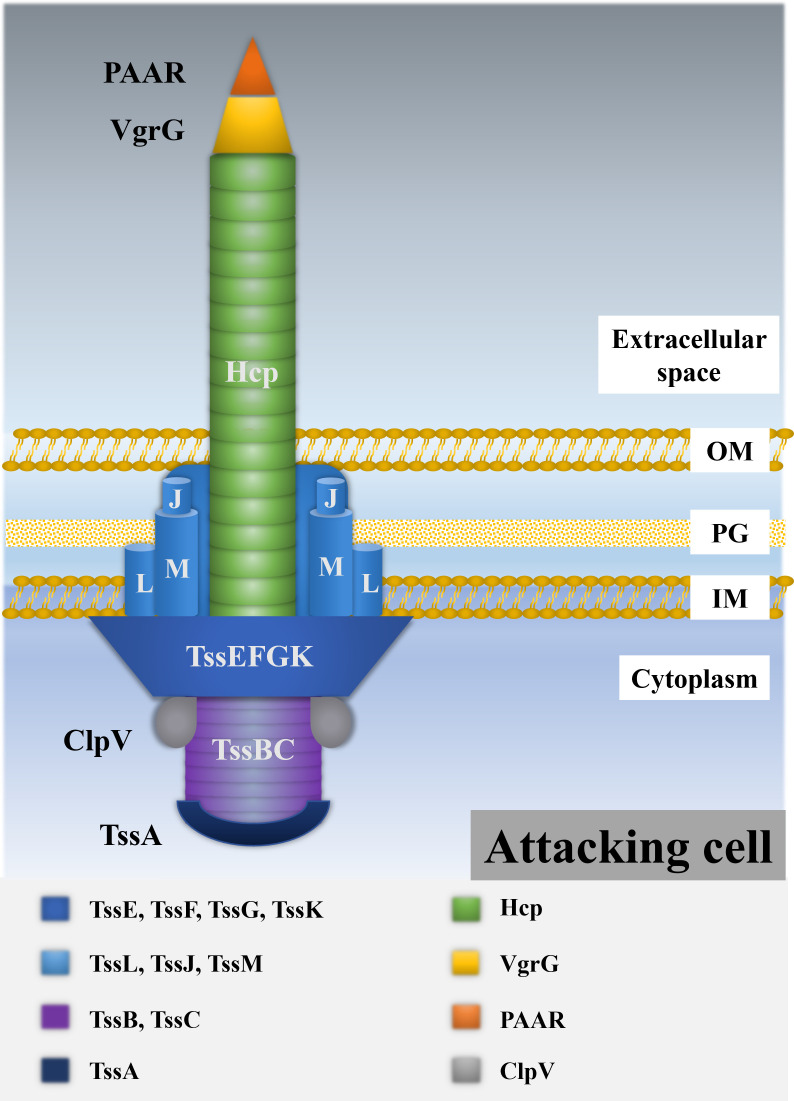
Schematic representation of the T6SS basic structure. Abbreviations: IM, inner membrane; OM, outer membrane; PG, peptidoglycan.

The T6SS is a conserved nanomachine with an inverted phage tail-like structure embedded in the bacterial cell membrane (Fig. 1). The structural core of the T6SS is typically encoded by a conserved cluster of 13 essential genes, referred to as *tssA* through *tssM* (type VI subunit/secretion genes) (23). In addition to these core components, a set of accessory genes—often termed “*tag*” (type VI associated genes)—such as *tagA*, *tagE*, *tagF*, *tagG*, *tagH*, *tagJ*, and *tagL*, contribute to the modulation and regulation of T6SS assembly. Furthermore, certain gene products of unknown function may endow the system with novel functional capacities in specific bacterial species (24). Structurally, the T6SS resembles an inverted contractile bacteriophage tail, composed of a central tubular structure formed by stacked hexameric rings of Hcp (hemolysin coregulated protein; also known as TssD), capped by a trimer of VgrG (valine-glycine repeat protein G; TssI), and further sharpened by the PAAR (proline-alanine-alanine-arginine repeat-containing) domain protein (TagD), which forms the spike tip (25). In *V. parahaemolyticus* T6SS1, however, the canonical PAAR domain is absent and functionally substituted by a PAAR-like protein with a DUF4150 domain, termed PIPY, which integrates into the spike complex (26, 27).

Encasing the inner tube is a contractile sheath made of TssB and TssC proteins arranged in a helical formation (23–25). This sheath-tube complex is assembled from a baseplate composed of the TssE, TssF, TssG, and TssK proteins (the TssEFGK complex), which nucleates tail polymerization within the cytoplasm (24, 28). The baseplate, in turn, anchors to the inner membrane via a transmembrane complex comprising TssL, TssM, and TssJ (collectively the TssLMJ complex), where TssL and TssM are inner membrane proteins, and TssJ is an outer membrane lipoprotein. This membrane complex is also responsible for linking the baseplate to the sheath (24, 28, 29). Additionally, TssA, a structural component of the system, is thought to facilitate T6SS biogenesis by orchestrating the coordinated assembly of baseplate, sheath, and tube elements (30, 31).

## T6SS GENE CLUSTERS OF *V. PARAHAEMOLYTICUS*

**Fig 2:**
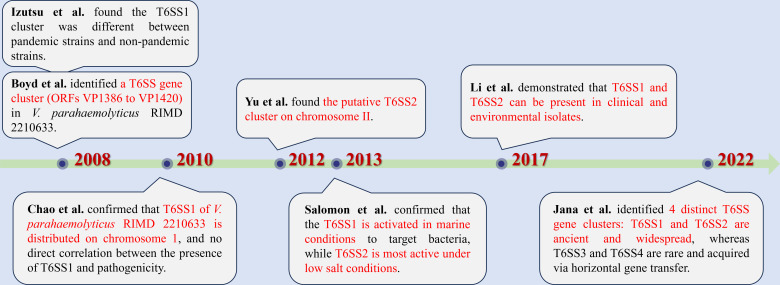
Discovery of T6SS gene clusters of *V. parahaemolyticus*.

The discovery of T6SS gene clusters in *V. parahaemolyticus* has unfolded through several pivotal research stages (Fig. 2). The initial breakthrough was made by Boyd et al., who utilized a four-way BLAST approach to analyze the genome of *V. parahaemolyticus* strain RIMD2210633, an O3:K6 serotype isolate collected in Japan in 1996. This analysis led to the identification of a T6SS gene cluster (>10 kb) spanning loci VP1386 to VP1420 (22). Around the same time, microarray-based comparisons between pandemic and non-pandemic strains revealed notable divergence in the T6SS1 region (32). Building on this, Chao et al. localized T6SS1 to chromosome I of the RIMD2210633 strain and observed that nearly 80% of pandemic isolates carried the complete T6SS1 locus. In contrast, around 70% of pathogenic strains contained only a subset of T6SS1 genes—specifically VP1390, VP1401, VP1405, VP1409, and VP1418—while nearly half of the non-pathogenic strains harbored only partial components of the system (33). Subsequently, Yu et al. identified a distinct T6SS cluster, termed T6SS2, encoded on chromosome II (8). Functional insights provided by Salomon et al. demonstrated that T6SS1 of *V. parahaemolyticus* RIMD2210633 is specifically activated under marine-like salinity and functions primarily in antibacterial interactions, whereas T6SS2 is more active under low-salt conditions and is regulated through distinct environmental cues (34). Li et al. later confirmed the co-occurrence of T6SS1 and T6SS2 in both clinical and environmental isolates of *V. parahaemolyticus* (35). Expanding the scope, Jana et al. conducted a large-scale genomic analysis involving 1,727 *V*. *parahaemolyticus* genomes. Their findings revealed the presence of four distinct T6SS loci. Among these, T6SS1 and T6SS2 were found to be evolutionarily ancient and widely distributed across the species, whereas T6SS3 and T6SS4 appeared to be recently acquired via horizontal gene transfer (HGT) and were present at low frequencies—0.8% and 1.8%, respectively—within the analyzed genomes (36). At present, it has been found that most *V. parahaemolyticus* isolates carry one or two T6SSs, and T6SS1 is encoded by a gene cluster that is predominantly found in pathogenic isolates. In contrast, T6SS2 is found in all *V. parahaemolyticus* isolates.

**Fig 3:**
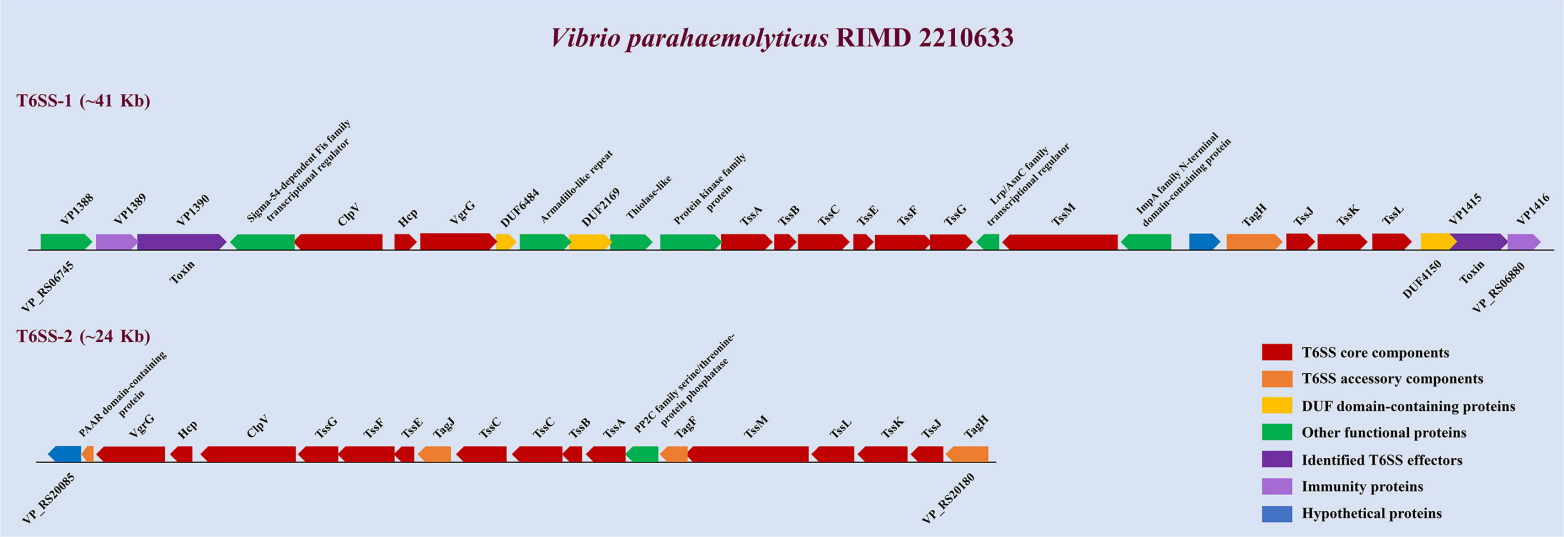
Schematic representation of the two T6SS gene clusters in *V. parahaemolyticus* RIMD 2210633. Core components of the T6SSs are shown in red; accessory components are shown in orange; DUF-containing proteins are represented in yellow; identified T6SS effectors (VP1390, VP1415) and immunity proteins (VP1389, VP1416) are shown in purple and lavender, respectively; other functional proteins in the two T6SSs are represented in green; and hypothetical proteins are shown in blue.

This review presents the two fully characterized T6SS gene clusters of *V. parahaemolyticus*, as illustrated in Fig. 3. T6SS1 and T6SS2 are encoded on chromosome I and chromosome II, respectively. The T6SS1 cluster spans about 41 kb (VP_RS06745 to VP_RS06880) and encodes 14 conserved core structural components. In contrast, the 24 kb T6SS2 cluster (VP_RS20085 to VP_RS20180) comprises 14 core genes along with four accessory genes: *tagF*, *tagH*, *tagJ*, and a gene encoding PAAR domain-containing protein. Both clusters have been identified in a wide range of clinical and environmental isolates of *V. parahaemolyticus* (37).

## EFFECTORS OF THE *V. PARAHAEMOLYTICUS* T6SS

**Fig 4:**
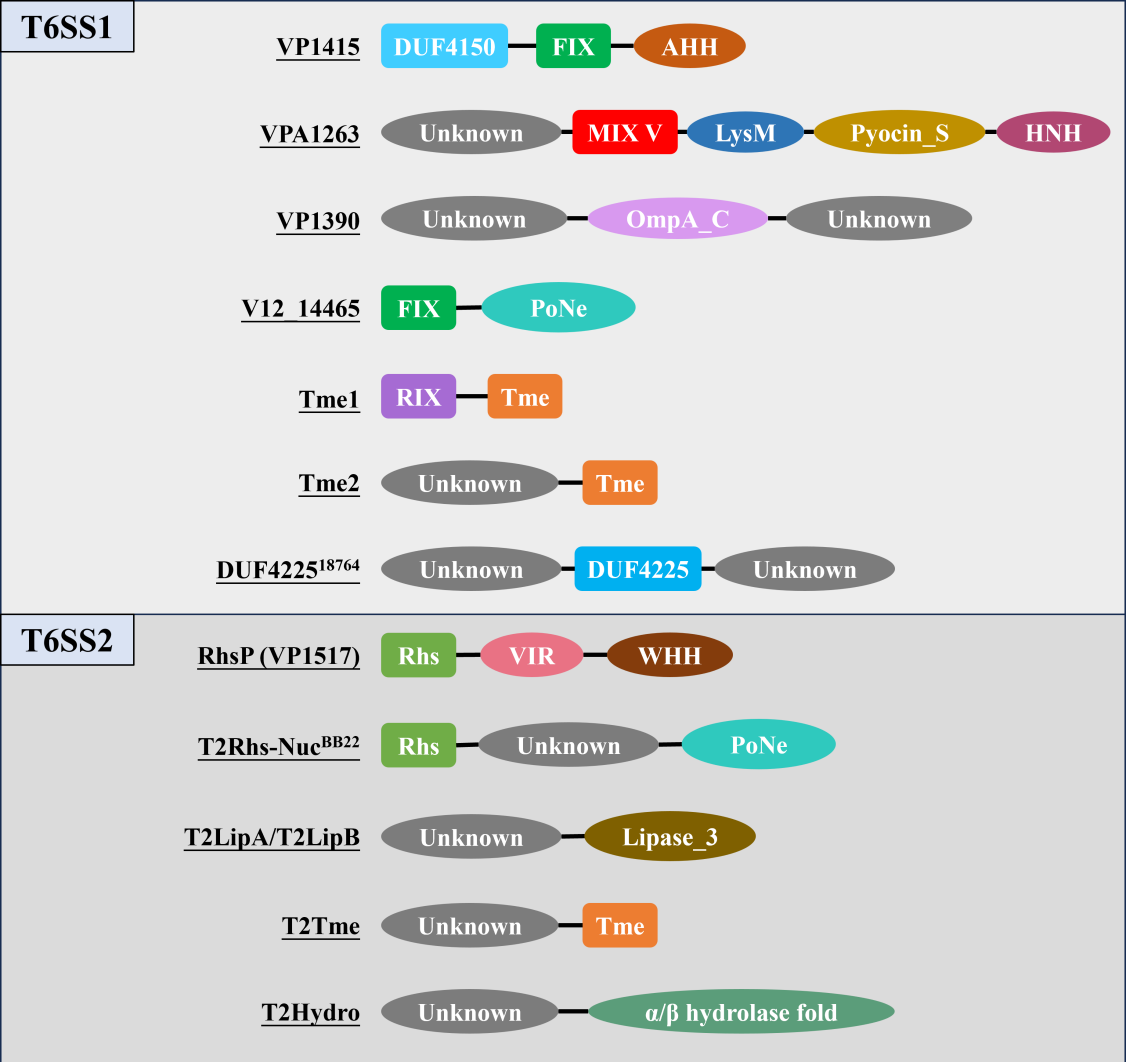
*Vibrio parahaemolyticus* T6SS effector repertoires. The figure shows the currently identified T6SS1 and T6SS2 effectors with conserved or established functional domains. Identified conserved domains: MIX V, FIX, RIX, DUF4150, DUF4225, and Rhs. Additional domains of known function: LysM, Pyocin_S; PoNe, Tme, HNH, AHH, VIR, WHH, Lipase_3, OmpA_C, and α/β hydrolase fold. Please note that the protein domains are schematic and not drawn to scale.

In *V. parahaemolyticus*, T6SS1 is highly prevalent and acts predominantly as an antibacterial system, enhancing survival and competitive fitness in marine environments. T6SS2, which is conserved across nearly all strains, plays integral roles in interbacterial competition and host cell adhesion, supporting its essential function in the bacterial lifecycle (8, 34, 37, 38). By contrast, it is hypothesized that T6SS3 specializes in anti-eukaryotic activity, while T6SS4 may contribute additional antibacterial functions; however, both systems are rare and together are present in only about 3% of analyzed *V. parahaemolyticus* genomes (36). The biological activity of T6SSs is closely tied to the repertoire of effector proteins they deliver. These effectors are critical for enabling *V. parahaemolyticus* to outcompete other bacterial species and persist under environmental stresses (36, 39, 40). This review synthesizes the current repertoire of experimentally validated and bioinformatically predicted T6SS effectors in *V. parahaemolyticus*, as summarized in Tables 1 and 2 and illustrated in Fig. 4.

**TABLE 1:** Identified T6SS1 effectors of *Vibrio parahaemolyticus*

Predicted effector name/locus	Known/predicted domain	Known/predicted activity	Strain	Reference(s)
VP1415	PAAR-like (DUF4150); FIX; AHH	Nuclease	RIMD 2210633	(27, 41)
VPA1263	MIX V; LysM; Pyocin_S; HNH nuclease	DNase	RIMD 2210633	
VP1390	OmpA_C	Cell lysis	RIMD 2210633	(42)
V12_14465	FIX; PoNe/DNase toxin	Nuclease	12-297/B	(43)
Tme1	RIX; Tme	Disrupting membrane integrity	BB22OP	(40, 44)
Tme2	Tme	Disrupting membrane integrity	T9109	(40)
DUF4225^18764^	DUF4225	Cell lysis	CFSAN018764	(36)

**TABLE 2:** Identified T6SS2 effectors of *Vibrio parahaemolyticus*

Predicted effector name	Known/predicted domain	Known/predicted activity	Strain(s)	Reference(s)
RhsP/T2Rhs-Nuc^RIMD^	Rhs repeats; VIR; WHH	Nuclease	RIMD 2210633	(45, 46)
T2Rhs-Nuc^BB22^	Rhs repeats; PoNe	Nuclease	BB22OP	(46)
T2Hydro	α/β hydrolase-fold	α/β hydrolase	RIMD 2210633	
T2LipA	Lipase_3	Lipase	BB22OP	
T2LipB	Lipase_3	Lipase	RIMD 2210633, BB22OP	
T2Tme	Tme	Disrupting membrane integrity	BB22OP	
T2Unkwn^BB22^	Unknown	Unknown	BB22OP	

### T6SS1-dependent effectors

T6SS1 mediates the secretion of a diverse suite of antibacterial effectors, most of which target bacterial cell envelope structures or nucleic acids, thereby disrupting cellular integrity in competing microbes. These effectors often contain conserved domains such as MIX, FIX, RIX, DUF4150, and DUF4225 (Table 1; Fig. 4). The MIX, FIX, and RIX domains are especially notable as molecular signatures for effector identification, typically located at the N-terminus and linked to C-terminal toxic domains, which confer specific antibacterial activities.

#### MIX domain effector: VPA1263

The MIX (Marker for type sIX effectors) domain is a conserved motif frequently located near T6SS gene clusters and fused to diverse bactericidal domains, such as nucleases, pore-forming toxins, phospholipases, peptidoglycan hydrolases, and proteases (41, 47, 48). Bioinformatic analyses indicate its prevalence in *Vibrionaceae*, with most species carrying one or two MIX-effectors in their genomes (47). In *V. parahaemolyticus* RIMD2210633, a MIX-containing effector (VPA1263) and a MIX-containing co-effector (VP1388) have been extensively characterized:

VP1388, located within the T6SS1 gene cluster (Fig. 3), was initially identified by Salomon et al. as a T6SS1-secreted effector based on comparative proteomics and its possession of an MIX I domain (41). Further studies by Dar et al. revealed that VP1388 operates within a distinct “binary effector module,” where it directly interacts with VP1390, and both are co-loaded onto the VgrG1 spike protein and rely on each other for successful secretion through T6SS1. VP1390 exerts periplasm-targeted toxicity, leading to observable cellular deformation and eventual lysis (42). Although initially described as an autonomous effector, VP1388 is now understood to serve as a tether that connects the toxin, VP1390, to the T6SS spike, possibly to VgrG. VPA1263, present in a limited subset of *V. parahaemolyticus* isolates, carries a MIX V domain and a LysM (Lysin Motif) domain at its N-terminal region, followed by a Pyocin_S domain and an HNH nuclease domain at the C-terminus. The N-terminal domains (MIX V and LysM) are necessary for T6SS1-dependent secretion, while the C-terminal domains mediate DNase activity, contributing to its antibacterial function (41, 49).

#### FIX domain effectors: VP1415 and V12_14465

The FIX (Found in type sIX effector) domain is a conserved N-terminal motif recognized as a key marker for T6SS-linked effector proteins. This domain is typically fused to various C-terminal toxin domains or uncharacterized extensions (43). Two FIX-domain effectors have been identified in *V. parahaemolyticus*, each representing distinct structural and functional properties:

V12_14465, identified in strain 12-297/B, comprises an N-terminal FIX domain directly linked to a C-terminal PoNe (polymorphic nuclease effector) domain, consistent with its role in mediating nuclease-based antibacterial toxicity through the T6SS apparatus. The PoNe domain belongs to the PD-(D/E)xK phosphodiesterase superfamily and functions as a DNase that targets DNA (43). Additionally, the PoNe domain has been identified across multiple bacterial toxin delivery pathways, including type V, type VI, and type VII secretion systems (43). Another effector containing a FIX domain, VP1415, is encoded within the T6SS1 gene cluster of *V. parahaemolyticus* strain RIMD2210633 (Fig. 3). This protein exemplifies a multifunctional effector, featuring an N-terminal PAAR-like domain (DUF4150), a central FIX domain, and a predicted AHH nuclease domain at the C-terminus, the latter of which is believed to be responsible for its DNase-mediated antibacterial activity (41).

#### Tme effectors and the RIX domain

Tme (type VI membrane disrupting effector) effectors (e.g., Tme1 and Tme2) constitute a class defined by a conserved C-terminal domain that exerts toxicity within the periplasm by disrupting bacterial membrane integrity (40). The N-terminal domain of Tme effectors remained elusive until further studies revealed that Tme1 possesses a novel RIX (aRginine-rich type sIX) domain at its N-terminus. The RIX domain is broadly conserved across the *Vibrionaceae* family and is consistently localized at the N-terminus of diverse C-terminal extensions, which may exhibit antibacterial or anti-eukaryotic activities, or alternatively function as loading platforms for cargo effectors. Critically, this RIX domain is both necessary and sufficient for T6SS1-mediated secretion of Tme1 in *V. parahaemolyticus* BB22OP. The identification of the RIX domain has significantly expanded the repertoire of known T6SS effectors and provides a powerful, family-specific marker for discovering novel effectors in *V. parahaemolyticus* (44).

The MIX, FIX, and RIX domains are key molecular signatures for classifying T6SS effectors (41, 43, 44). Although MIX and FIX are widely distributed across T6SS-encoding bacteria, the RIX domain exhibits a more restricted, lineage-specific distribution that is predominantly confined to the *Vibrionaceae*. Of particular relevance to *V. parahaemolyticus*, the RIX domain plays a critical role in Tme1 secretion and is uniquely conserved and consistently positioned at the N-terminus of effectors within *Vibrionaceae*, making it an effective tool for effector discovery. Likewise, the widespread presence of MIX and FIX domains provides valuable markers for expanding the known effector repertoire of this pathogen. These conserved signatures continue to significantly expand the catalog of known T6SS effector families.

#### Additional characterized effectors

As discussed in the context of the “binary effector module” involving VP1388, VP1390 is an antibacterial effector encoded within the T6SS1 cluster of *V. parahaemolyticus* strain RIMD2210633 (Fig. 3). It contains an OmpA_C domain that mediates peptidoglycan binding (50). Dar et al. showed that VP1390 exerts its toxicity in the periplasm, ultimately leading to bacterial cell lysis (42). Another antibacterial determinant, the DUF4225 domain, has been identified as a broadly distributed toxic domain associated with multiple secretion systems, including the T6SS. In *V. parahaemolyticus* strain CFSAN018764, a DUF4225-containing protein (DUF4225^18764^), located downstream of *hcp1b*, was shown to function as a T6SS1 antibacterial effector. Upon translocation into the periplasm of target cells, it triggers cell lysis (36).

### T6SS2-dependent effectors

Tang et al. identified RhsP (VP1517) as a T6SS2-delivered effector in *V. parahaemolyticus* RIMD2210633. RhsP exhibits hallmark polymorphic toxin features, containing an Rhs (Rearrangement hotspot) domain and a C-terminal WHH nuclease motif with conserved catalytic residues responsible for its DNA-targeting activity (45). Additionally, RhsP possesses a C-terminal VgrG2-interacting region (VIR), which undergoes conformational changes following autoproteolysis. This conformational shift facilitates RhsP dimerization, a process essential for its targeting and toxic action against competitor bacteria (45).

In a complementary study, Tchelet et al. employed proteomic analyses to define the T6SS2 secretome of two *V. parahaemolyticus* strains, BB22OP and RIMD2210633, identifying a suite of secreted antibacterial effectors, all of which are encoded outside the primary T6SS2 gene cluster (46). Among these, T2Rhs-Nuc and T2LipB were found to be conserved across multiple strains, suggesting their inclusion within the core T6SS2 secretome. Additional effectors, such as T2LipA, T2Tme, T2Hydro, and T2Unkwn, were strain-specific, indicating their role as part of an accessory effector arsenal. Notably, T2Rhs-Nuc corresponds to the RhsP effector characterized by Tang et al., confirming its conserved function and identity across independent studies (45, 46).

### Other effectors

Beyond the experimentally confirmed T6SS1- and T6SS2-secreted effectors, Jana et al. extensively cataloged putative *V. parahaemolyticus* effectors harboring either known or predicted toxin domains based on a systematic analysis of all available RefSeq *V. parahaemolyticus* genomes (Table 3). Genomic localization analyses have revealed that numerous putative effector genes are embedded within or adjacent to the T6SS3 and T6SS4 gene clusters, or are genomically linked to loci encoding key structural components of the T6SS, such as PAAR, Hcp, and VgrG proteins (36). Collectively, these discoveries substantially broaden the known effector repertoire of conserved T6SSs, encompassing novel effectors with uncharacterized activities and functions not previously linked to T6SS machinery. Nevertheless, the specific domains and precise functions of these effectors necessitate further experimental validation.

**TABLE 3:** Predicted T6SS effectors of *Vibrio parahaemolyticus^a^*

Example accession no.	Example gene locus	Found in T6SS/module type	Known/predicted domain	Known/predicted activity
WP_029843206.1	LPR98_RS07710	T6SS3	Unknown	Unknown
WP_042762256.1	PO80_RS02570	T6SS4	Unknown	Unknown
WP_193237005.1	JHS79_RS25745	T6SS4	Lip2	Lipase
WP_065771704.1	AKH09_RS09365	PAAR+VgrG+Hcp	Unknown	Unknown
WP_238790300.1	K6U37_RS14065	PAAR+VgrG	NucA/B	Nuclease
WP_020841305.1	H9J98_RS02420	PAAR	Ntox15	Nuclease
WP_083135234.1	GPY55_RS17385	PAAR	NUC	Nuclease
WP_102591288.1	C1T08_RS26340	PAAR	Unknown	Unknown
WP_102591220.1	C1S85_RS24675	PAAR	Tme	Membrane disrupting
WP_102591225.1	C1S85_RS24700	PAAR	Unknown	Unknown
WP_238789479.1	K6U37_RS04455	PAAR	Unknown	Unknown
WP_129147717.1	EGL73_RS17180	VgrG	Unknown	Unknown
WP_029857615.1	B5C30_RS14465	VgrG	PoNe	DNase
WP_238790289.1	K6U37_RS13990	VgrG	Unknown	Unknown
WP_086585359.1	JHS88_RS14235	Hcp	DUF4225	Cell lysis
WP_195433156.1	K6U37_RS12660	Hcp	Unknown	Unknown
WP_228085946.1	IB292_RS21975	Hcp	Unknown	Unknown

^*a*^ The table is adapted from Jana et al. (36).

## REGULATORY FACTORS OF THE *V. PARAHAEMOLYTICUS* T6SS

**Fig 5:**
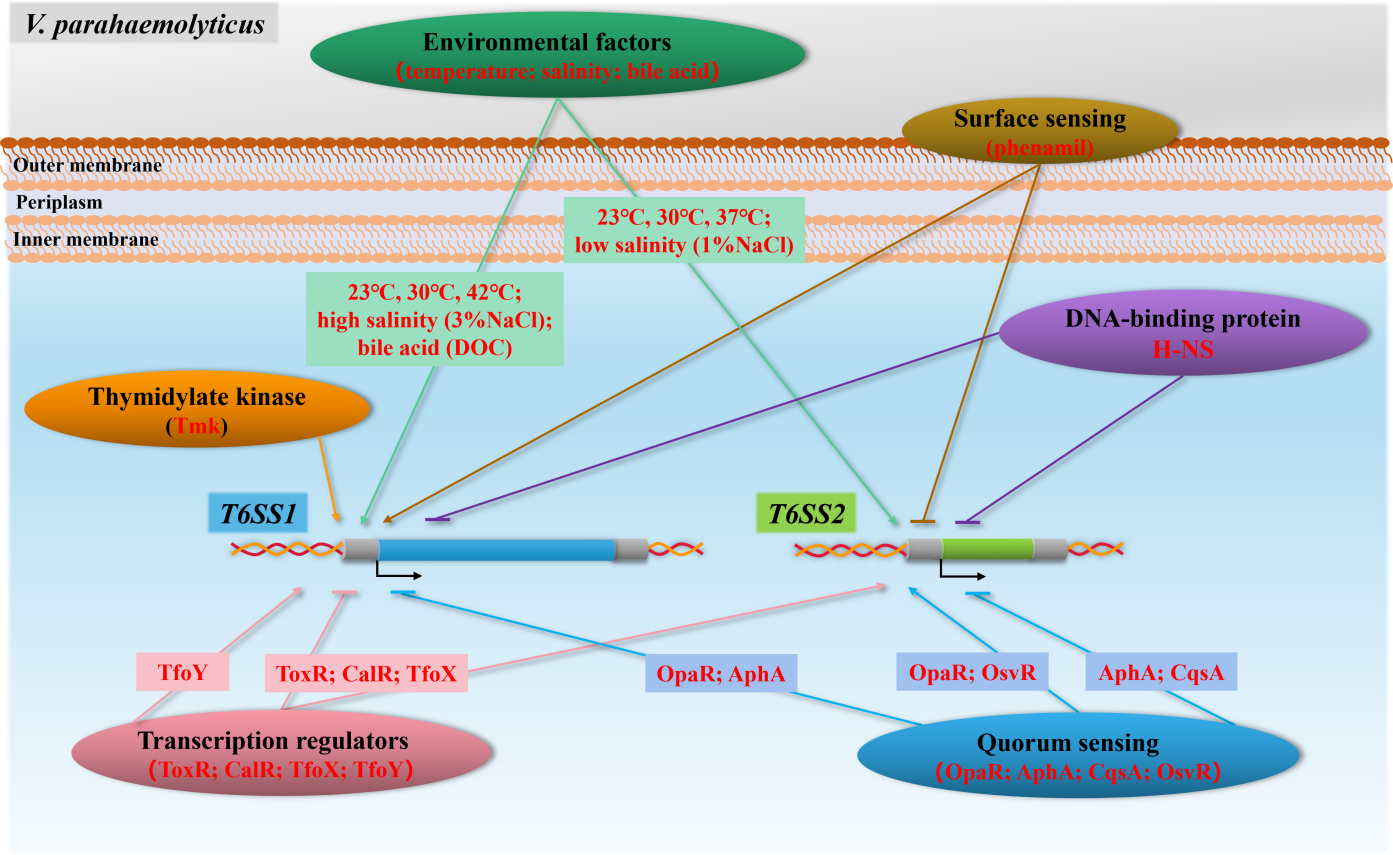
Model of the *V. parahaemolyticus* T6SS regulatory network. T6SS1 is induced under warm marine-like conditions (23°C, 30°C, 42°C), high salinity (3%NaCl), surface sensing (via phenamil), regulator TfoY, and thymidylate kinase (Tmk). It is inhibited by DNA-binding protein H-NS, transcription regulators ToxR, CalR, and TfoX, as well as the quorum-sensing core regulators OpaR and AphA. T6SS2 is activated under both cold and warm temperatures (23°C, 30°C, 37°C) in low-salinity (1%NaCl) conditions and positively regulated by ToxR, CalR, TfoX, OpaR, and a new quorum-sensing transcriptional factor QsvR, whereas it is repressed by surface sensing, H-NS, AphA, and CqsA-introduced quorum sensing.

The regulation of type VI secretion systems in *V. parahaemolyticus* is intricately governed by a multitude of environmental stimuli and regulatory proteins. Accumulating evidence indicates that the expression and activity of both T6SS1 and T6SS2 are influenced by the quorum-sensing network, DNA-binding protein H-NS, surface sensing, and a range of environmental growth conditions—including variations in temperature, salinity, and the presence of bile acids (34, 37, 51, 52) (Fig. 5).

### Environmental factors

Environmental cues serve as key modulators of T6SS activation in *V. parahaemolyticus*. Salomon et al. systematically investigated how these factors affect the expression and secretion of hallmark T6SS structural proteins, Hcp1 and Hcp2, in strain RIMD2210633 (34). Their study demonstrated that T6SS1 and T6SS2 are differentially regulated by temperature and salt concentration. Specifically, Hcp1 of T6SS1 was expressed at 23°C and 30°C, but not at 37°C, under both high-salinity (3% NaCl) and low-salinity (1% NaCl) conditions, with higher expression levels observed in the former. However, its secretion was limited to high-salinity conditions at 30°C and, to a lesser extent, under low salinity at 23°C. Conversely, Hcp2, associated with T6SS2, was expressed across all tested temperatures (23°C, 30°C, and 37°C), but its secretion occurred only under low-salinity conditions at 23°C and 30°C, with slightly greater secretion observed at 23°C (34). These results suggest that T6SS1 exhibits peak activity and antibacterial function under high salinity and warm temperatures, whereas T6SS2 remains active across both cold and warm temperatures in low-salinity conditions. Moreover, T6SS1 expression in *V. parahaemolyticus* has been shown to be upregulated at 42°C, supporting earlier observations by Salomon et al. regarding the system’s responsiveness to elevated environmental temperatures commonly encountered in warm marine climates (53). Furthermore, Schiffmann et al. demonstrated that secondary bile acids activate T6SS1 in *V. parahaemolyticus*, with deoxycholate (DOC) specifically potentiating the system’s antibacterial activity to confer a competitive advantage during interbacterial competition (51).

### Surface sensing

Surface sensing—the bacterial capacity to detect and respond to physical contact with solid substrates—triggers complex regulatory pathways that alter gene expression and behavior (54). In *V. parahaemolyticus*, surface sensing differentially influences the activity of T6SS1 and T6SS2. Prior studies examined the expression and secretion dynamics of Hcp1 and Hcp2 in *V. parahaemolyticus* exposed to increasing concentrations of phenamil, a polar flagellar motor inhibitor used to mimic surface-associated conditions during bacterial growth in liquid cultures (40, 55). Phenamil treatment led to increased expression and secretion of Hcp1. Conversely, while total Hcp2 expression was unaffected, its secretion was significantly inhibited by phenamil. These findings suggest that surface sensing activates T6SS1 while concurrently repressing T6SS2 secretion, reflecting a divergent regulatory response to environmental contact stimuli.

### Quorum sensing system

Quorum sensing (QS) is a cell-density-dependent communication mechanism involving the synthesis, release, and perception of extracellular signaling molecules known as autoinducers. This system is extensively distributed among *Vibrio* species and plays a key role in coordinating collective bacterial behaviors (56, 57). The involvement of QS in regulating T6SS expression in *V. parahaemolyticus* has been well established. Zhang et al. reported that T6SS1 expression peaks during the mid-exponential growth phase, specifically when the culture’s optical density at 600 nm (OD_600_) reaches 0.6 to 0.8, highlighting the system’s tight linkage to QS-mediated population-level regulation (58).

OpaR and AphA serve as key regulators of QS, with OpaR being highly expressed at high bacterial densities and AphA at low bacterial densities (59). At low population densities, the quorum-sensing regulator LuxO remains active, leading to the suppression of OpaR expression and the activation of AphA. As cell density increases, LuxO activity is inhibited, allowing OpaR to accumulate while repressing AphA (56). Functionally, OpaR has been shown to repress the transcription of *hcp1*, thereby negatively regulating T6SS1, while concurrently acting as a positive regulator of T6SS2 (37, 52). In contrast, AphA acts as a repressor of both T6SS1 and T6SS2 expression (37, 52, 58, 60).

More recently, a newly identified quorum-sensing transcription factor, QsvR, was found to directly promote T6SS2 expression by binding to promoter-proximal DNA regions (61). In a study employing tandem mass tag (TMT)-based proteomic profiling, Wu et al. reported that deletion of *cqsA*—the gene encoding the synthase of the quorum-sensing molecule 3-hydroxyundecan-4-one—resulted in enhanced T6SS2 production in *V. parahaemolyticus* (62). Gene expression analyses showed that T6SS2 transcripts were significantly upregulated in the Δ*cqsA* mutant, but markedly downregulated in both the Δ*opaR* single mutant and the Δ*cqsA*Δ*opaR* double mutant. These observations suggest that OpaR plays a predominant role in the regulation of T6SS2 by the CqsA-introduced quorum sensing (CIQS) system. Furthermore, the study also confirmed that CIQS suppresses T6SS2 production and T6SS2-mediated cell adhesion to HeLa cells through an OpaR-dependent pathway (62).

### DNA-binding protein H-NS

The histone-like nucleoid structure DNA-binding protein (H-NS) is a nucleic acid binding protein and can bind to AT-rich DNA sequences, thereby inhibiting gene transcription (63). In *V. parahaemolyticus*, H-NS has been characterized as a repressor of both T6SS1 and T6SS2. It inhibits T6SS2 by directly binding to its promoter regions, while T6SS1 repression occurs through downregulation of *hcp1* expression and secretion under various environmental contexts (64). Importantly, activation of surface sensing and exposure to high-salinity conditions have been shown to relieve H-NS-mediated repression of T6SS1 (65).

### Transcription regulators

CalR, a LysR-type transcriptional regulator, was initially identified as a repressor of swarming motility and T3SS1 gene expression in *V. parahaemolyticus* (66, 67). The T6SS2 system in *V. parahaemolyticus* has been shown to promote bacterial adherence to HeLa epithelial cells (8). Zhang et al. further identified CalR as a transcriptional activator of T6SS2-mediated adhesion. CalR enhances T6SS2 gene expression by directly binding to the promoter regions of three putative operons (VPA1027-1024, VPA1043-1028, and VPA1044-1046) located within the T6SS2 genomic locus (58). Additionally, ToxR, a membrane-associated regulator of virulence, has been implicated in the repression of T6SS1 activity through cooperative interactions with the quorum-sensing regulators AphA and OpaR (58).

In bacteria, particularly within the *Vibrio* genus, TfoX and TfoY function as master transcriptional regulators orchestrating genetic transformation and virulence factor expression (68). Metzger et al. demonstrated that the TfoX‐ and TfoY‐mediated signaling pathways are mostly conserved in diverse *Vibrio* species and important for signal-specific T6SS induction. Their findings revealed that TfoX specifically induces T6SS2 expression, while TfoY is responsible for activating T6SS1 and promoting antibacterial activity (69). Supporting this, Ben-Yaakov et al. confirmed that TfoY functions as a key positive regulator of T6SS1 in *V. parahaemolyticus* and is both necessary and sufficient for system activation. In contrast, overexpression of TfoX was found to suppress T6SS1 activity (39).

#### Others

Further investigations by Ben-Yaakov et al. identified thymidylate kinase (Tmk)—an enzyme that catalyzes the phosphorylation of thymidine 5′-monophosphate (dTMP) to thymidine 5′-diphosphate (dTDP) as part of the *de novo* and salvage pathways of thymidine 5′-triphosphate (dTTP) biosynthesis—as an additional factor required for the activation of T6SS1 (39).

## CONCLUSIONS

As a globally significant foodborne pathogen, *Vibrio parahaemolyticus* presents a serious threat to both public health and the aquaculture sector. Its type VI secretion systems constitute a key component of its adaptive and pathogenic toolkit, facilitating survival in competitive marine environments and enhancing its ability to antagonize other microbial species. This review has provided a detailed overview of the T6SS’s structural components, assembly processes, effector proteins, and regulatory mechanisms. The two Type VI secretion systems in *V. parahaemolyticus*, T6SS1 and T6SS2, are functionally and regulatory distinct, each responding to specific environmental cues, such as temperature shifts, salinity levels, surface sensing, quorum sensing, DNA-binding protein, and dedicated regulatory factors.

Although considerable insights have been gained into the operation of these systems, several pivotal questions remain unanswered. For instance, beyond their competitive antibacterial roles, might *V. parahaemolyticus* T6SSs also provide mutualistic benefits to neighboring cells under certain conditions? Given the diversity of secreted effectors, what environmental or physiological triggers govern their expression and release? Are all effectors deployed concurrently, or is there a selective, context-dependent regulatory mechanism that determines which effectors are secreted? Furthermore, could additional, uncharacterized effectors remain undiscovered? The structural domains and secretion pathways of many known effectors are still poorly understood. Equally important is understanding how external signals are sensed and integrated at the molecular level to modulate T6SS activity. The broader regulatory architecture of *V. parahaemolyticus* T6SSs—particularly components that operate outside traditional regulatory circuits—has yet to be fully mapped. Addressing these outstanding challenges will not only deepen our understanding of T6SS biology but may also inform new strategies to mitigate the public health risks and foodborne threats posed by *V. parahaemolyticus*.

## Data Availability

No data were used for the research described in the article.
